# (4-Amino­pyridine-κ*N* ^1^)(2,2′-bi­pyridine-κ^2^ *N*,*N*′)(2,2′:6′,2′′-terpyridine-κ^3^ *N*,*N*′,*N*′′)ruthenium(II) bis­(hexa­fluorido­phosphate) unknown solvate

**DOI:** 10.1107/S241431462100287X

**Published:** 2021-03-23

**Authors:** Carly R. Reed, Robert N. Garner, William W. Brennessel

**Affiliations:** a Department of Chemistry and Biochemistry, The College at Brockport, SUNY, Brockport, NY 14420, USA; b Department of Chemistry and Biochemistry, University of the Incarnate Word, San Antonio, Texas 78209, USA; c Department of Chemistry, University of Rochester, Rochester, NY 14627, USA; Benemérita Universidad Autónoma de Puebla, México

**Keywords:** crystal structure, ruthenium, bi­pyridine, terpyridine, amino­pyridine

## Abstract

The title compound, [Ru(C_5_H_6_N_2_)(C_10_H_8_N_2_)(C_15_H_11_N_3_)](PF_6_)_2_ solvent, crystallizes in the triclinic space group *P*  with one dicationic Ru complex, two PF_6_^−^ anions, and undefined solvent in the asymmetric unit. The cation and anions are linked *via* N—H⋯F hydrogen bonding. One PF_6_^−^ anion is disordered over two positions, with occupancies 0.634 (8) and 0.366 (8). The solvent, which is located in channels in the crystal, is highly disordered. Reflection contributions from the solvent were added to the calculated structure factors using the *SQUEEZE* routine [Spek (2015) *Acta Cryst.* C**71**, 9–18] of the program *PLATON*, which determined there to be 59 electrons in 264 Å^3^ treated this way per unit cell. Because the exact identity and amount of solvent were unknown, no solvent was included in the atom list or mol­ecular formula.

## Abstract 2

The ruthenium dication and hexa­fluorido­phosphate anions of the title salt are linked by N—H⋯F hydrogen bonds at the amino group of the substituted pyridine ring. The Ru—N bond lengths show the expected range.

## Graphical abstract

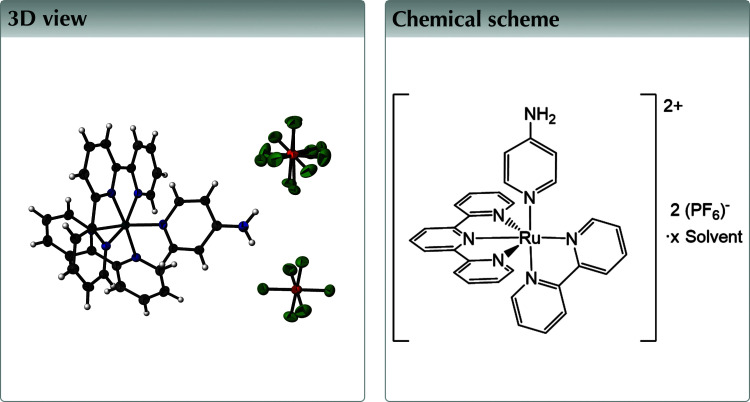

## Structure description

**Figure 1:**
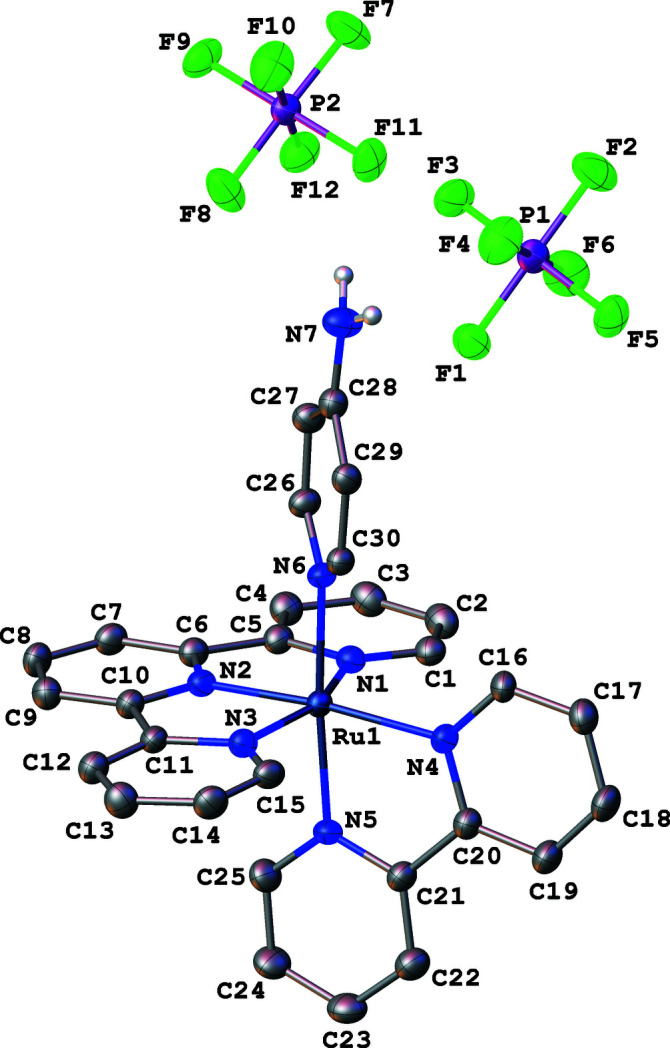
Anisotropic displacement ellipsoid plot drawn at the 50% probability level. Only the major component of the disordered PF_6_ anion and only the NH_2_ hydrogen atoms are displayed. Highly disordered solvent, located in channels parallel to [10 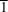 ], is not shown (see *Refinement details*).

**Figure 2:**
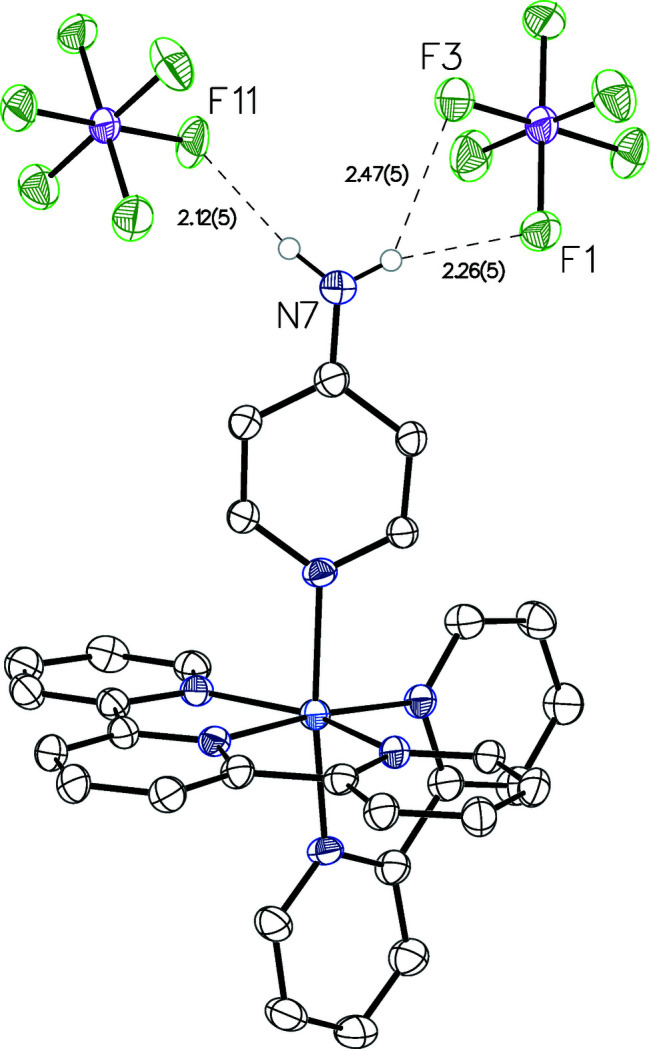
Anisotropic displacement ellipsoid plot drawn at the 50% probability level. Only the major component of the disordered PF_6_^−^ anion and only the NH_2_ hydrogen atoms are displayed. To one anion the N—H⋯F hydrogen bonding is bifurcated (see Table 2 ▸ for additional metrical details).

The reported complex was explored previously to determine the impact of pyridine substitution on mol­ecular excited states (Vu *et al.*, 2016 ▸). The complex (Fig. 1 ▸) has the expected structure, very similar to the unsubstituted pyridine analog (Hecker *et al.*, 1991 ▸). All Ru—N bond lengths fall within the expected range (Table 1 ▸), with the longest Ru—N bond occurring to the amino­pyridine and the shortest to the terpyridine central nitro­gen. The bi­pyridine Ru—N bond *cis* to the amino­pyridine is elongated due to steric inter­actions with the pyridine ring. In the crystal, N—H⋯F hydrogen bonding links the cation to the anions (Table 2 ▸ and Fig. 2 ▸).

## Synthesis and crystallization

The complex was synthesized according to previously published procedures (Vu *et al.* 2016 ▸). Single crystals suitable for X-ray diffraction were obtained by dissolving the complex in a 1:1 ratio, by volume, of acetonitrile and methanol. A few drops of chloroform were added to this mixture. The solvent mixture was then layered with half as much diethyl ether. The solution was stored in a 248 K freezer in an open test tube for two weeks until small needle-shaped dark-red crystals formed.

## Refinement

Crystal data, data collection and structure refinement details are summarized in Table 3 ▸. Hexa­fluorido­phosphate anion P2/F7⋯F12 is modeled as disordered over two positions [occupancies: 0.634 (8) for P2/F7⋯F12 and 0.366 (8) for P2′/F7′⋯F12′]. Analogous bond lengths and angles were restrained to be similar. Anisotropic displacement parameters for proximal atoms were restrained to be similar and restrained toward the expected motion relative to bond direction.

Reflection contributions from highly disordered solvent, located in channels parallel to [10 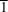 ], were added to the calculated structure factors using the *SQUEEZE* routine (Spek, 2015 ▸) of the program *PLATON*, which determined there to be 59 electrons in 264 Å^3^ treated this way per unit cell. Because the exact identity and amount of solvent were unknown, no solvent was included in the atom list or mol­ecular formula. Thus, all calculated qu­anti­ties that derive from the mol­ecular formula [*e.g*., *F*(000), density, mol­ecular weight, *etc*.] are known to be inaccurate.

The maximum residual peak of 0.89 e Å^−3^ and the deepest hole of −1.46 e Å^−3^ are found 1.07 and 0.83 Å from atoms N3 and Ru1, respectively.

## Supplementary Material

Crystal structure: contains datablock(s) I, global. DOI: 10.1107/S241431462100287X/bh4060sup1.cif

Structure factors: contains datablock(s) I. DOI: 10.1107/S241431462100287X/bh4060Isup2.hkl

CCDC reference: 2071159

Additional supporting information:  crystallographic information; 3D view; checkCIF report

## Figures and Tables

**Table 1:** Selected bond lengths (Å)

Ru1—N1	2.080 (2)	Ru1—N4	2.093 (2)
Ru1—N2	1.962 (2)	Ru1—N5	2.058 (2)
Ru1—N3	2.079 (2)	Ru1—N6	2.113 (2)

**Table 2:** Hydrogen-bond geometry (Å, °)

*D*—H⋯*A*	*D*—H	H⋯*A*	*D*⋯*A*	*D*—H⋯*A*
N7—H7*A*⋯F11	0.90 (5)	2.12 (5)	3.008 (5)	169 (4)
N7—H7*A*⋯F11′	0.90 (5)	2.07 (5)	2.901 (8)	154 (4)
N7—H7*B*⋯F1	0.85 (5)	2.26 (5)	3.061 (4)	159 (4)
N7—H7*B*⋯F3	0.85 (5)	2.47 (5)	3.156 (4)	139 (4)

**Table 3:** Experimental details

Crystal data	
Chemical formula	[Ru(C_5_H_6_N_2_)(C_10_H_8_N_2_)(C_15_H_11_N_3_)](PF_6_)_2_
*M*_r_	874.58
Crystal system, space group	Triclinic, *P* 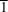
Temperature (K)	100
*a*, *b*, *c* (Å)	11.6594 (4), 13.4335 (2), 13.7219 (3)
α, β, γ (°)	64.764 (2), 69.337 (3), 86.320 (2)
*V* (Å^3^)	1809.56 (9)
*Z*	2
Radiation type	Cu *K*α
μ (mm^−1^)	5.21
Crystal size (mm)	0.21 × 0.05 × 0.04

Data collection	
Diffractometer	Rigaku Oxford Diffraction XtaLAB Synergy, Dualflex, HyPix
Absorption correction	Multi-scan (*CrysAlis PRO*; Rigaku OD, 2018 ▸)
*T*_min_, *T*_max_	0.604, 1.000
No. of measured, independent and observed [*I* > 2σ(*I*)] reflections	37534, 7587, 6954
*R*_int_	0.069
(sin θ/λ)_max_ (Å^−1^)	0.634

Refinement	
*R*[*F* ^2^ > 2σ(*F* ^2^)], *wR*(*F* ^2^), *S*	0.044, 0.124, 1.09
No. of reflections	7587
No. of parameters	535
No. of restraints	57
H-atom treatment	H atoms treated by a mixture of independent and constrained refinement
Δρ_max_, Δρ_min_ (e Å^−3^)	0.89, −1.46

Computer programs: *CrysAlis PRO* (Rigaku OD, 2018 ▸), ShelXT (Sheldrick, 2015*a* ▸), *SHELXL* (Sheldrick, 2015*b* ▸), and*OLEX2* (Dolomanov *et al.*, 2009 ▸).

## full crystallographic data

### Crystal data

**Table:**

[Ru(C_5_H_6_N_2_)(C_10_H_8_N_2_)(C_15_H_11_N_3_)](PF_6_)_2_	*Z* = 2
*M**_r_* = 874.58	*F*(000) = 872
Triclinic, *P*1	*D*_x_ = 1.605 Mg m^−^^3^
*a* = 11.6594 (4) Å	Cu *K*α radiation, λ = 1.54184 Å
*b* = 13.4335 (2) Å	Cell parameters from 21227 reflections
*c* = 13.7219 (3) Å	θ = 3.7–77.5°
α = 64.764 (2)°	µ = 5.21 mm^−^^1^
β = 69.337 (3)°	*T* = 100 K
γ = 86.320 (2)°	Needle, red
*V* = 1809.56 (9) Å^3^	0.21 × 0.05 × 0.04 mm

### Data collection

**Table:**

Rigaku Oxford Diffraction XtaLAB Synergy, Dualflex, HyPix diffractometer	7587 independent reflections
Radiation source: micro-focus sealed X-ray tube, PhotonJet (Cu) X-ray Source	6954 reflections with *I* > 2σ(*I*)
Mirror monochromator	*R*_int_ = 0.069
ω scans	θ_max_ = 77.9°, θ_min_ = 3.7°
Absorption correction: multi-scan (CrysAlisPro; Rigaku OD, 2018)	*h* = −14→14
*T*_min_ = 0.604, *T*_max_ = 1.000	*k* = −16→16
37534 measured reflections	*l* = −14→17

### Refinement

**Table:**

Refinement on *F*^2^	Primary atom site location: dual
Least-squares matrix: full	Secondary atom site location: difference Fourier map
*R*[*F*^2^ > 2σ(*F*^2^)] = 0.044	Hydrogen site location: mixed
*wR*(*F*^2^) = 0.124	H atoms treated by a mixture of independent and constrained refinement
*S* = 1.09	*w* = 1/[σ^2^(*F*_o_^2^) + (0.081*P*)^2^ + 0.8329*P*] where *P* = (*F*_o_^2^ + 2*F*_c_^2^)/3
7587 reflections	(Δ/σ)_max_ = 0.001
535 parameters	Δρ_max_ = 0.89 e Å^−^^3^
57 restraints	Δρ_min_ = −1.46 e Å^−^^3^

### Special details

**Table:**

Refinement. Reflection contributions from highly disordered solvent were fixed and added to the calculated structure factors using the SQUEEZE routine of program Platon (Spek, 2015), which determined there to be 59 electrons in 264 Å^3^ treated this way per unit cell. Because the exact identity and amount of solvent were unknown, no solvent was included in the atom list or molecular formula. Thus all calculated quantities that derive from the molecular formula (e.g., F(000), density, molecular weight, etc.) are known to be incorrect.Hexafluorophophate anion P2/F7-F12 is modeled as disordered over two positions (0.634 (8):0.366 (8)). Analogous bond lengths and angles were restrained to be similar. Anisotropic displacement parameters for proximal atoms were restrained to be similar and restrained toward the expected motion relative to bond direction.The NH_2_ hydrogen atoms were located in a difference Fourier map and refined freely. The remaining hydrogen atoms were given riding models: C—H = 0.93 Å and *U*_iso_(H) = 1.2*U*_eq_(carrier C).

### Fractional atomic coordinates and isotropic or equivalent isotropic displacement parameters (Å^2^)

**Table:**

	*x*	*y*	*z*	*U*_iso_*/*U*_eq_	Occ. (<1)
Ru1	0.37269 (2)	0.38839 (2)	0.81766 (2)	0.02018 (9)	
P1	1.10072 (8)	0.73218 (7)	0.26920 (7)	0.02873 (18)	
P2	0.7331 (5)	1.0900 (5)	0.3394 (5)	0.0252 (5)	0.634 (8)
F7	0.7674 (5)	1.1703 (4)	0.2054 (4)	0.0474 (13)	0.634 (8)
F8	0.6970 (5)	1.0075 (4)	0.4726 (3)	0.0460 (12)	0.634 (8)
F9	0.6916 (7)	1.1905 (6)	0.3720 (8)	0.0407 (16)	0.634 (8)
F10	0.8698 (3)	1.1133 (5)	0.3305 (5)	0.0496 (13)	0.634 (8)
F11	0.7770 (5)	0.9895 (3)	0.3067 (4)	0.0411 (10)	0.634 (8)
F12	0.5973 (3)	1.0663 (3)	0.3487 (4)	0.0398 (10)	0.634 (8)
P2'	0.7491 (9)	1.0940 (9)	0.3397 (9)	0.0252 (5)	0.366 (8)
F7'	0.7352 (9)	1.1218 (10)	0.2193 (8)	0.050 (2)	0.366 (8)
F8'	0.7637 (9)	1.0637 (9)	0.4585 (6)	0.064 (3)	0.366 (8)
F9'	0.6634 (14)	1.1874 (13)	0.3561 (16)	0.057 (4)	0.366 (8)
F10'	0.8666 (7)	1.1817 (8)	0.2721 (7)	0.053 (2)	0.366 (8)
F11'	0.8379 (11)	1.0021 (6)	0.3211 (7)	0.063 (3)	0.366 (8)
F12'	0.6336 (9)	1.0051 (9)	0.4044 (8)	0.075 (4)	0.366 (8)
F1	1.0213 (2)	0.65559 (17)	0.40258 (17)	0.0374 (4)	
F2	1.1806 (2)	0.80744 (19)	0.13693 (18)	0.0517 (6)	
F3	1.0644 (2)	0.83963 (17)	0.29106 (19)	0.0451 (5)	
F4	1.2197 (2)	0.7291 (2)	0.3027 (2)	0.0479 (5)	
F5	1.1364 (2)	0.62302 (17)	0.24975 (18)	0.0409 (5)	
F6	0.9824 (2)	0.7333 (2)	0.2368 (2)	0.0475 (5)	
N1	0.2568 (2)	0.4706 (2)	0.7296 (2)	0.0218 (5)	
N2	0.2868 (2)	0.46862 (19)	0.9086 (2)	0.0218 (5)	
N3	0.4523 (2)	0.3333 (2)	0.9430 (2)	0.0220 (5)	
N4	0.4476 (2)	0.2897 (2)	0.7314 (2)	0.0224 (5)	
N5	0.2485 (2)	0.2490 (2)	0.9140 (2)	0.0228 (5)	
N6	0.5148 (2)	0.5200 (2)	0.7125 (2)	0.0221 (5)	
N7	0.8026 (3)	0.7730 (2)	0.4900 (2)	0.0323 (6)	
H7A	0.788 (4)	0.839 (4)	0.443 (4)	0.045 (12)*	
H7B	0.874 (5)	0.756 (4)	0.462 (4)	0.039 (11)*	
C1	0.2434 (3)	0.4657 (2)	0.6385 (3)	0.0254 (6)	
H1	0.291460	0.420904	0.607439	0.030*	
C2	0.1609 (3)	0.5247 (3)	0.5887 (3)	0.0306 (6)	
H2	0.153700	0.519234	0.525753	0.037*	
C3	0.0893 (3)	0.5919 (3)	0.6342 (3)	0.0307 (6)	
H3	0.034405	0.633183	0.601418	0.037*	
C4	0.1007 (3)	0.5969 (3)	0.7288 (3)	0.0275 (6)	
H4	0.052755	0.641032	0.760924	0.033*	
C5	0.1837 (3)	0.5359 (2)	0.7757 (2)	0.0236 (6)	
C6	0.2013 (3)	0.5350 (2)	0.8771 (2)	0.0229 (5)	
C7	0.1376 (3)	0.5906 (2)	0.9419 (3)	0.0275 (6)	
H7	0.079962	0.637559	0.920137	0.033*	
C8	0.1610 (3)	0.5754 (3)	1.0395 (3)	0.0286 (6)	
H8	0.118928	0.612239	1.083467	0.034*	
C9	0.2473 (3)	0.5051 (3)	1.0718 (3)	0.0274 (6)	
H9	0.262188	0.493652	1.137817	0.033*	
C10	0.3104 (3)	0.4525 (2)	1.0044 (2)	0.0234 (5)	
C11	0.4040 (3)	0.3739 (2)	1.0248 (2)	0.0230 (5)	
C12	0.4403 (3)	0.3416 (2)	1.1177 (3)	0.0275 (6)	
H12	0.405843	0.369343	1.172197	0.033*	
C13	0.5288 (3)	0.2674 (3)	1.1306 (3)	0.0305 (6)	
H13	0.553485	0.244645	1.193696	0.037*	
C14	0.5795 (3)	0.2280 (3)	1.0476 (3)	0.0294 (6)	
H14	0.640121	0.179610	1.053191	0.035*	
C15	0.5383 (3)	0.2622 (2)	0.9562 (3)	0.0253 (6)	
H15	0.571622	0.234576	0.901436	0.030*	
C16	0.5486 (3)	0.3166 (3)	0.6349 (2)	0.0262 (6)	
H16	0.589513	0.387274	0.599341	0.031*	
C17	0.5944 (3)	0.2441 (3)	0.5865 (3)	0.0303 (6)	
H17	0.665195	0.265426	0.520469	0.036*	
C18	0.5334 (3)	0.1395 (3)	0.6375 (3)	0.0341 (7)	
H18	0.562727	0.088974	0.606993	0.041*	
C19	0.4278 (3)	0.1115 (3)	0.7348 (3)	0.0332 (7)	
H19	0.384625	0.041956	0.769832	0.040*	
C20	0.3864 (3)	0.1874 (2)	0.7802 (2)	0.0258 (6)	
C21	0.2743 (3)	0.1641 (2)	0.8831 (3)	0.0259 (6)	
C22	0.2002 (3)	0.0650 (3)	0.9452 (3)	0.0328 (7)	
H22	0.219654	0.008042	0.922482	0.039*	
C23	0.0973 (3)	0.0506 (3)	1.0408 (3)	0.0341 (7)	
H23	0.047101	−0.016087	1.083761	0.041*	
C24	0.0702 (3)	0.1377 (3)	1.0714 (3)	0.0342 (7)	
H24	0.001050	0.130683	1.135126	0.041*	
C25	0.1470 (3)	0.2343 (3)	1.0063 (3)	0.0305 (6)	
H25	0.127937	0.292519	1.027142	0.037*	
C26	0.4942 (3)	0.6264 (2)	0.6595 (3)	0.0248 (6)	
H26	0.412847	0.642386	0.672401	0.030*	
C27	0.5858 (3)	0.7132 (2)	0.5873 (3)	0.0263 (6)	
H27	0.565308	0.784856	0.554318	0.032*	
C28	0.7101 (3)	0.6927 (2)	0.5640 (2)	0.0251 (6)	
C29	0.7319 (3)	0.5828 (2)	0.6237 (2)	0.0241 (6)	
H29	0.812223	0.564980	0.615131	0.029*	
C30	0.6346 (3)	0.5016 (2)	0.6948 (2)	0.0218 (5)	
H30	0.652158	0.429826	0.732977	0.026*	

### Atomic displacement parameters (Å^2^)

**Table:**

	*U*^11^	*U*^22^	*U*^33^	*U*^12^	*U*^13^	*U*^23^
Ru1	0.02185 (13)	0.01787 (13)	0.01976 (13)	0.00208 (8)	−0.00411 (9)	−0.00992 (9)
P1	0.0302 (4)	0.0258 (4)	0.0281 (4)	0.0078 (3)	−0.0069 (3)	−0.0133 (3)
P2	0.0259 (14)	0.0224 (5)	0.0259 (4)	0.0062 (8)	−0.0056 (7)	−0.0125 (3)
F7	0.053 (3)	0.037 (2)	0.0266 (17)	0.016 (2)	−0.0008 (17)	−0.0028 (18)
F8	0.056 (3)	0.045 (2)	0.0261 (17)	0.0199 (19)	−0.0098 (16)	−0.0117 (15)
F9	0.040 (3)	0.030 (2)	0.057 (4)	0.0088 (19)	−0.012 (2)	−0.028 (2)
F10	0.0279 (18)	0.064 (3)	0.068 (3)	0.0084 (18)	−0.0137 (18)	−0.041 (3)
F11	0.055 (3)	0.0336 (19)	0.043 (2)	0.0238 (19)	−0.0195 (19)	−0.0247 (15)
F12	0.0327 (18)	0.039 (2)	0.052 (2)	0.0043 (14)	−0.0122 (15)	−0.0257 (18)
P2'	0.0259 (14)	0.0224 (5)	0.0259 (4)	0.0062 (8)	−0.0056 (7)	−0.0125 (3)
F7'	0.052 (5)	0.063 (6)	0.042 (4)	0.014 (4)	−0.023 (4)	−0.025 (5)
F8'	0.068 (6)	0.092 (7)	0.029 (3)	0.036 (5)	−0.019 (4)	−0.025 (4)
F9'	0.056 (8)	0.059 (7)	0.060 (6)	0.039 (6)	−0.020 (5)	−0.034 (4)
F10'	0.046 (4)	0.051 (5)	0.056 (5)	−0.021 (3)	0.001 (3)	−0.030 (4)
F11'	0.072 (6)	0.033 (4)	0.043 (4)	0.028 (4)	0.008 (4)	−0.004 (3)
F12'	0.063 (6)	0.074 (6)	0.059 (5)	−0.034 (5)	0.024 (4)	−0.034 (5)
F1	0.0420 (11)	0.0331 (10)	0.0298 (9)	0.0064 (8)	−0.0089 (8)	−0.0107 (8)
F2	0.0529 (14)	0.0400 (12)	0.0336 (10)	0.0117 (10)	0.0055 (9)	−0.0085 (9)
F3	0.0476 (13)	0.0297 (10)	0.0489 (12)	0.0081 (9)	−0.0023 (10)	−0.0215 (9)
F4	0.0347 (11)	0.0551 (14)	0.0661 (14)	0.0075 (10)	−0.0201 (10)	−0.0358 (12)
F5	0.0560 (13)	0.0330 (10)	0.0431 (11)	0.0180 (9)	−0.0223 (10)	−0.0231 (9)
F6	0.0473 (13)	0.0495 (13)	0.0487 (12)	0.0148 (10)	−0.0271 (11)	−0.0175 (10)
N1	0.0215 (11)	0.0203 (11)	0.0208 (11)	0.0004 (9)	−0.0053 (9)	−0.0081 (9)
N2	0.0223 (12)	0.0184 (11)	0.0212 (11)	0.0011 (9)	−0.0038 (9)	−0.0087 (9)
N3	0.0223 (12)	0.0198 (11)	0.0222 (11)	0.0036 (9)	−0.0055 (9)	−0.0098 (9)
N4	0.0251 (12)	0.0195 (11)	0.0231 (11)	0.0032 (9)	−0.0066 (9)	−0.0115 (9)
N5	0.0258 (12)	0.0178 (11)	0.0196 (11)	−0.0023 (9)	−0.0035 (9)	−0.0065 (9)
N6	0.0242 (12)	0.0172 (11)	0.0208 (11)	−0.0016 (9)	−0.0037 (9)	−0.0077 (9)
N7	0.0269 (14)	0.0248 (13)	0.0331 (14)	0.0013 (11)	−0.0031 (11)	−0.0077 (11)
C1	0.0249 (14)	0.0240 (14)	0.0253 (13)	−0.0012 (11)	−0.0053 (11)	−0.0114 (11)
C2	0.0287 (16)	0.0364 (17)	0.0266 (14)	−0.0019 (13)	−0.0094 (12)	−0.0132 (13)
C3	0.0280 (15)	0.0338 (16)	0.0280 (14)	0.0027 (12)	−0.0106 (12)	−0.0110 (13)
C4	0.0223 (14)	0.0300 (15)	0.0282 (15)	0.0032 (11)	−0.0057 (11)	−0.0137 (12)
C5	0.0210 (13)	0.0228 (13)	0.0252 (13)	0.0013 (10)	−0.0044 (11)	−0.0118 (11)
C6	0.0214 (13)	0.0213 (13)	0.0238 (13)	0.0029 (10)	−0.0044 (10)	−0.0108 (11)
C7	0.0276 (15)	0.0229 (14)	0.0304 (15)	0.0052 (11)	−0.0071 (12)	−0.0132 (12)
C8	0.0333 (16)	0.0257 (15)	0.0281 (14)	0.0075 (12)	−0.0071 (12)	−0.0167 (12)
C9	0.0308 (16)	0.0273 (15)	0.0243 (14)	0.0031 (12)	−0.0080 (12)	−0.0130 (12)
C10	0.0270 (14)	0.0205 (13)	0.0227 (13)	0.0026 (11)	−0.0070 (11)	−0.0109 (11)
C11	0.0269 (14)	0.0187 (12)	0.0213 (13)	−0.0001 (11)	−0.0044 (11)	−0.0098 (10)
C12	0.0311 (16)	0.0244 (14)	0.0275 (14)	0.0041 (12)	−0.0084 (12)	−0.0136 (12)
C13	0.0301 (16)	0.0316 (16)	0.0297 (15)	0.0081 (13)	−0.0109 (12)	−0.0138 (13)
C14	0.0324 (16)	0.0233 (14)	0.0320 (15)	0.0087 (12)	−0.0117 (13)	−0.0122 (12)
C15	0.0258 (14)	0.0194 (13)	0.0264 (14)	0.0038 (11)	−0.0043 (11)	−0.0104 (11)
C16	0.0319 (15)	0.0256 (14)	0.0219 (13)	−0.0004 (12)	−0.0052 (11)	−0.0141 (11)
C17	0.0301 (16)	0.0340 (16)	0.0269 (14)	0.0021 (12)	−0.0043 (12)	−0.0180 (13)
C18	0.0406 (19)	0.0294 (16)	0.0353 (16)	0.0036 (13)	−0.0066 (14)	−0.0223 (14)
C19	0.0411 (18)	0.0268 (15)	0.0318 (15)	0.0002 (13)	−0.0061 (14)	−0.0179 (13)
C20	0.0294 (15)	0.0237 (14)	0.0247 (13)	0.0026 (11)	−0.0068 (12)	−0.0132 (11)
C21	0.0295 (15)	0.0214 (13)	0.0261 (14)	0.0021 (11)	−0.0070 (12)	−0.0121 (11)
C22	0.0355 (17)	0.0260 (15)	0.0340 (16)	0.0007 (13)	−0.0071 (13)	−0.0144 (13)
C23	0.0308 (17)	0.0282 (16)	0.0343 (16)	−0.0057 (13)	−0.0056 (13)	−0.0091 (13)
C24	0.0260 (16)	0.0334 (17)	0.0330 (16)	−0.0041 (13)	0.0009 (12)	−0.0136 (14)
C25	0.0282 (16)	0.0300 (16)	0.0275 (14)	0.0017 (12)	−0.0008 (12)	−0.0144 (12)
C26	0.0242 (14)	0.0203 (13)	0.0275 (14)	0.0030 (11)	−0.0062 (11)	−0.0109 (11)
C27	0.0276 (15)	0.0215 (13)	0.0259 (14)	0.0045 (11)	−0.0070 (11)	−0.0092 (11)
C28	0.0278 (15)	0.0239 (14)	0.0239 (13)	0.0032 (11)	−0.0077 (11)	−0.0119 (11)
C29	0.0254 (14)	0.0221 (14)	0.0237 (13)	0.0060 (11)	−0.0066 (11)	−0.0111 (11)
C30	0.0224 (13)	0.0197 (12)	0.0215 (12)	0.0024 (10)	−0.0043 (10)	−0.0103 (10)

### Geometric parameters (Å, º)

**Table:**

Ru1—N1	2.080 (2)	C4—H4	0.9300
Ru1—N2	1.962 (2)	C4—C5	1.381 (4)
Ru1—N3	2.079 (2)	C5—C6	1.472 (4)
Ru1—N4	2.093 (2)	C6—C7	1.389 (4)
Ru1—N5	2.058 (2)	C7—H7	0.9300
Ru1—N6	2.113 (2)	C7—C8	1.388 (5)
P1—F1	1.608 (2)	C8—H8	0.9300
P1—F2	1.596 (2)	C8—C9	1.391 (4)
P1—F3	1.598 (2)	C9—H9	0.9300
P1—F4	1.601 (2)	C9—C10	1.380 (4)
P1—F5	1.604 (2)	C10—C11	1.482 (4)
P1—F6	1.589 (2)	C11—C12	1.372 (4)
P2—F7	1.597 (6)	C12—H12	0.9300
P2—F8	1.591 (6)	C12—C13	1.391 (4)
P2—F9	1.593 (7)	C13—H13	0.9300
P2—F10	1.596 (6)	C13—C14	1.388 (4)
P2—F11	1.598 (6)	C14—H14	0.9300
P2—F12	1.585 (6)	C14—C15	1.386 (4)
P2'—F7'	1.596 (11)	C15—H15	0.9300
P2'—F8'	1.573 (11)	C16—H16	0.9300
P2'—F9'	1.595 (11)	C16—C17	1.383 (4)
P2'—F10'	1.583 (11)	C17—H17	0.9300
P2'—F11'	1.606 (11)	C17—C18	1.381 (5)
P2'—F12'	1.577 (11)	C18—H18	0.9300
N1—C1	1.342 (4)	C18—C19	1.383 (5)
N1—C5	1.375 (4)	C19—H19	0.9300
N2—C6	1.356 (4)	C19—C20	1.387 (4)
N2—C10	1.362 (4)	C20—C21	1.474 (4)
N3—C11	1.382 (4)	C21—C22	1.381 (4)
N3—C15	1.345 (4)	C22—H22	0.9300
N4—C16	1.348 (4)	C22—C23	1.379 (5)
N4—C20	1.361 (4)	C23—H23	0.9300
N5—C21	1.359 (4)	C23—C24	1.388 (5)
N5—C25	1.343 (4)	C24—H24	0.9300
N6—C26	1.350 (4)	C24—C25	1.372 (5)
N6—C30	1.356 (4)	C25—H25	0.9300
N7—H7A	0.90 (5)	C26—H26	0.9300
N7—H7B	0.85 (5)	C26—C27	1.382 (4)
N7—C28	1.342 (4)	C27—H27	0.9300
C1—H1	0.9300	C27—C28	1.405 (4)
C1—C2	1.388 (5)	C28—C29	1.408 (4)
C2—H2	0.9300	C29—H29	0.9300
C2—C3	1.384 (5)	C29—C30	1.378 (4)
C3—H3	0.9300	C30—H30	0.9300
C3—C4	1.380 (4)		
			
N1—Ru1—N4	99.51 (9)	C3—C2—H2	120.5
N1—Ru1—N6	92.43 (9)	C2—C3—H3	120.5
N2—Ru1—N1	79.62 (10)	C4—C3—C2	119.1 (3)
N2—Ru1—N3	79.70 (10)	C4—C3—H3	120.5
N2—Ru1—N4	173.73 (9)	C3—C4—H4	120.2
N2—Ru1—N5	95.58 (10)	C3—C4—C5	119.7 (3)
N2—Ru1—N6	90.50 (10)	C5—C4—H4	120.2
N3—Ru1—N1	159.27 (10)	N1—C5—C4	121.6 (3)
N3—Ru1—N4	100.87 (9)	N1—C5—C6	114.8 (2)
N3—Ru1—N6	89.24 (10)	C4—C5—C6	123.5 (3)
N4—Ru1—N6	95.74 (10)	N2—C6—C5	113.4 (2)
N5—Ru1—N1	91.01 (10)	N2—C6—C7	119.7 (3)
N5—Ru1—N3	89.51 (10)	C7—C6—C5	126.8 (3)
N5—Ru1—N4	78.20 (10)	C6—C7—H7	120.4
N5—Ru1—N6	173.47 (9)	C8—C7—C6	119.2 (3)
F2—P1—F1	179.44 (13)	C8—C7—H7	120.4
F2—P1—F3	90.87 (12)	C7—C8—H8	119.9
F2—P1—F4	90.07 (14)	C7—C8—C9	120.2 (3)
F2—P1—F5	90.13 (12)	C9—C8—H8	119.9
F3—P1—F1	89.52 (11)	C8—C9—H9	120.4
F3—P1—F4	90.55 (13)	C10—C9—C8	119.1 (3)
F3—P1—F5	178.96 (12)	C10—C9—H9	120.4
F4—P1—F1	89.52 (13)	N2—C10—C9	120.0 (3)
F4—P1—F5	89.14 (12)	N2—C10—C11	112.7 (2)
F5—P1—F1	89.48 (11)	C9—C10—C11	127.3 (3)
F6—P1—F1	90.00 (12)	N3—C11—C10	115.0 (2)
F6—P1—F2	90.40 (14)	C12—C11—N3	121.4 (3)
F6—P1—F3	90.18 (13)	C12—C11—C10	123.6 (3)
F6—P1—F4	179.12 (13)	C11—C12—H12	120.0
F6—P1—F5	90.12 (13)	C11—C12—C13	120.0 (3)
F7—P2—F11	89.3 (4)	C13—C12—H12	120.0
F8—P2—F7	178.4 (5)	C12—C13—H13	120.6
F8—P2—F9	90.4 (5)	C14—C13—C12	118.8 (3)
F8—P2—F10	90.4 (4)	C14—C13—H13	120.6
F8—P2—F11	89.6 (4)	C13—C14—H14	120.6
F9—P2—F7	90.7 (5)	C15—C14—C13	118.8 (3)
F9—P2—F10	90.3 (4)	C15—C14—H14	120.6
F9—P2—F11	179.0 (5)	N3—C15—C14	123.0 (3)
F10—P2—F7	90.8 (4)	N3—C15—H15	118.5
F10—P2—F11	88.7 (4)	C14—C15—H15	118.5
F12—P2—F7	89.4 (4)	N4—C16—H16	118.5
F12—P2—F8	89.5 (4)	N4—C16—C17	123.1 (3)
F12—P2—F9	89.8 (4)	C17—C16—H16	118.5
F12—P2—F10	179.8 (6)	C16—C17—H17	120.5
F12—P2—F11	91.1 (3)	C18—C17—C16	119.0 (3)
F7'—P2'—F11'	88.4 (7)	C18—C17—H17	120.5
F8'—P2'—F7'	178.6 (9)	C17—C18—H18	120.7
F8'—P2'—F9'	90.1 (8)	C17—C18—C19	118.7 (3)
F8'—P2'—F10'	91.5 (7)	C19—C18—H18	120.7
F8'—P2'—F11'	90.3 (7)	C18—C19—H19	120.0
F8'—P2'—F12'	89.5 (8)	C18—C19—C20	119.9 (3)
F9'—P2'—F7'	91.3 (8)	C20—C19—H19	120.0
F9'—P2'—F11'	178.7 (11)	N4—C20—C19	121.5 (3)
F10'—P2'—F7'	88.9 (7)	N4—C20—C21	115.3 (2)
F10'—P2'—F9'	90.3 (9)	C19—C20—C21	123.2 (3)
F10'—P2'—F11'	88.5 (7)	N5—C21—C20	114.5 (3)
F12'—P2'—F7'	90.0 (7)	N5—C21—C22	121.5 (3)
F12'—P2'—F9'	91.0 (9)	C22—C21—C20	124.0 (3)
F12'—P2'—F10'	178.3 (9)	C21—C22—H22	120.0
F12'—P2'—F11'	90.2 (7)	C23—C22—C21	119.9 (3)
C1—N1—Ru1	128.8 (2)	C23—C22—H22	120.0
C1—N1—C5	117.9 (2)	C22—C23—H23	120.7
C5—N1—Ru1	113.24 (18)	C22—C23—C24	118.5 (3)
C6—N2—Ru1	118.85 (19)	C24—C23—H23	120.7
C6—N2—C10	121.7 (2)	C23—C24—H24	120.5
C10—N2—Ru1	119.36 (19)	C25—C24—C23	119.0 (3)
C11—N3—Ru1	113.22 (19)	C25—C24—H24	120.5
C15—N3—Ru1	128.9 (2)	N5—C25—C24	123.2 (3)
C15—N3—C11	117.9 (2)	N5—C25—H25	118.4
C16—N4—Ru1	127.1 (2)	C24—C25—H25	118.4
C16—N4—C20	117.8 (2)	N6—C26—H26	117.8
C20—N4—Ru1	115.11 (19)	N6—C26—C27	124.4 (3)
C21—N5—Ru1	116.80 (19)	C27—C26—H26	117.8
C25—N5—Ru1	125.2 (2)	C26—C27—H27	120.1
C25—N5—C21	117.9 (3)	C26—C27—C28	119.8 (3)
C26—N6—Ru1	123.5 (2)	C28—C27—H27	120.1
C26—N6—C30	115.5 (2)	N7—C28—C27	122.1 (3)
C30—N6—Ru1	120.99 (19)	N7—C28—C29	121.9 (3)
H7A—N7—H7B	113 (4)	C27—C28—C29	116.0 (3)
C28—N7—H7A	121 (3)	C28—C29—H29	119.9
C28—N7—H7B	119 (3)	C30—C29—C28	120.1 (3)
N1—C1—H1	118.6	C30—C29—H29	119.9
N1—C1—C2	122.7 (3)	N6—C30—C29	123.9 (3)
C2—C1—H1	118.6	N6—C30—H30	118.0
C1—C2—H2	120.5	C29—C30—H30	118.0
C3—C2—C1	119.0 (3)		
			
Ru1—N1—C1—C2	179.3 (2)	C5—C6—C7—C8	176.2 (3)
Ru1—N1—C5—C4	180.0 (2)	C6—N2—C10—C9	−0.6 (4)
Ru1—N1—C5—C6	0.0 (3)	C6—N2—C10—C11	178.0 (3)
Ru1—N2—C6—C5	0.8 (3)	C6—C7—C8—C9	0.0 (5)
Ru1—N2—C6—C7	178.6 (2)	C7—C8—C9—C10	1.1 (5)
Ru1—N2—C10—C9	−177.5 (2)	C8—C9—C10—N2	−0.8 (5)
Ru1—N2—C10—C11	1.0 (3)	C8—C9—C10—C11	−179.1 (3)
Ru1—N3—C11—C10	−3.0 (3)	C9—C10—C11—N3	179.8 (3)
Ru1—N3—C11—C12	176.3 (2)	C9—C10—C11—C12	0.6 (5)
Ru1—N3—C15—C14	−176.8 (2)	C10—N2—C6—C5	−176.2 (3)
Ru1—N4—C16—C17	177.3 (2)	C10—N2—C6—C7	1.7 (4)
Ru1—N4—C20—C19	−177.7 (2)	C10—C11—C12—C13	180.0 (3)
Ru1—N4—C20—C21	2.5 (3)	C11—N3—C15—C14	0.2 (4)
Ru1—N5—C21—C20	−2.7 (3)	C11—C12—C13—C14	0.5 (5)
Ru1—N5—C21—C22	177.0 (2)	C12—C13—C14—C15	−1.4 (5)
Ru1—N5—C25—C24	−176.6 (3)	C13—C14—C15—N3	1.0 (5)
Ru1—N6—C26—C27	−177.8 (2)	C15—N3—C11—C10	179.6 (3)
Ru1—N6—C30—C29	177.3 (2)	C15—N3—C11—C12	−1.2 (4)
N1—C1—C2—C3	0.2 (5)	C16—N4—C20—C19	1.8 (4)
N1—C5—C6—N2	−0.5 (4)	C16—N4—C20—C21	−178.0 (3)
N1—C5—C6—C7	−178.2 (3)	C16—C17—C18—C19	0.6 (5)
N2—C6—C7—C8	−1.4 (5)	C17—C18—C19—C20	−1.0 (5)
N2—C10—C11—N3	1.4 (4)	C18—C19—C20—N4	−0.3 (5)
N2—C10—C11—C12	−177.9 (3)	C18—C19—C20—C21	179.5 (3)
N3—C11—C12—C13	0.8 (5)	C19—C20—C21—N5	−179.7 (3)
N4—C16—C17—C18	1.0 (5)	C19—C20—C21—C22	0.7 (5)
N4—C20—C21—N5	0.1 (4)	C20—N4—C16—C17	−2.2 (4)
N4—C20—C21—C22	−179.5 (3)	C20—C21—C22—C23	179.7 (3)
N5—C21—C22—C23	0.1 (5)	C21—N5—C25—C24	1.2 (5)
N6—C26—C27—C28	0.9 (5)	C21—C22—C23—C24	0.7 (5)
N7—C28—C29—C30	−176.9 (3)	C22—C23—C24—C25	−0.5 (5)
C1—N1—C5—C4	−1.5 (4)	C23—C24—C25—N5	−0.4 (5)
C1—N1—C5—C6	178.5 (2)	C25—N5—C21—C20	179.4 (3)
C1—C2—C3—C4	−1.1 (5)	C25—N5—C21—C22	−1.0 (5)
C2—C3—C4—C5	0.6 (5)	C26—N6—C30—C29	−3.1 (4)
C3—C4—C5—N1	0.6 (5)	C26—C27—C28—N7	176.5 (3)
C3—C4—C5—C6	−179.4 (3)	C26—C27—C28—C29	−4.0 (4)
C4—C5—C6—N2	179.5 (3)	C27—C28—C29—C30	3.6 (4)
C4—C5—C6—C7	1.8 (5)	C28—C29—C30—N6	0.0 (4)
C5—N1—C1—C2	1.0 (4)	C30—N6—C26—C27	2.7 (4)

### Hydrogen-bond geometry (Å, º)

**Table:**

*D*—H···*A*	*D*—H	H···*A*	*D*···*A*	*D*—H···*A*
N7—H7*A*···F11	0.90 (5)	2.12 (5)	3.008 (5)	169 (4)
N7—H7*A*···F11′	0.90 (5)	2.07 (5)	2.901 (8)	154 (4)
N7—H7*B*···F1	0.85 (5)	2.26 (5)	3.061 (4)	159 (4)
N7—H7*B*···F3	0.85 (5)	2.47 (5)	3.156 (4)	139 (4)
